# An Approach to Robust INS/UWB Integrated Positioning for Autonomous Indoor Mobile Robots

**DOI:** 10.3390/s19040950

**Published:** 2019-02-23

**Authors:** Jianfeng Liu, Jiexin Pu, Lifan Sun, Zishu He

**Affiliations:** 1 School of Information Engineering, Henan University of Science and Technology, Luoyang 471023, China; liu_jfeng@163.com (J.L.); pjx@haust.edu.cn (J.P.); 2 School of Communication and Information Engineering, University of Electronic Science and Technology of China, Chengdu 611731, China; zshe@uestc.edu.cn

**Keywords:** indoor mobile robots, INS/UWB integrated positioning, Sage–Husa fuzzy adaptive filter, innovation contribution weight, outliers detection and correction

## Abstract

The key to successful positioning of autonomous mobile robots in complicated indoor environments lies in the strong anti-interference of the positioning system and accurate measurements from sensors. Inertial navigation systems (INS) are widely used for indoor mobile robots because they are not susceptible to external interferences and work properly, but the positioning errors may be accumulated over time. Thus ultra wideband (UWB) is usually adopted to compensate the accumulated errors due to its high ranging precision. Unfortunately, UWB is easily affected by the multipath effects and non-line-of-sight (NLOS) factor in complex indoor environments, which may degrade the positioning performance. To solve above problems, this paper proposes an effective system framework of INS/UWB integrated positioning for autonomous indoor mobile robots, in which our modeling approach is simple to implement and a Sage–Husa fuzzy adaptive filter (SHFAF) is proposed. Due to the favorable property (i.e., self-adaptive adjustment) of SHFAF, the difficult problem of time-varying noise in complex indoor environments is considered and solved explicitly. Moreover, outliers can be detected and corrected by the proposed sliding window estimation with fading coefficients. This facilitates the positioning performance improvement for indoor mobile robots. The benefits of what we propose are illustrated by not only simulations but more importantly experimental results.

## 1. Introduction

The positioning technology plays an important role in navigation for mobile robots and can be classified into the absolute positioning and relative positioning [1,2,3]. The former generally depends on reference nodes with known absolute locations, which is adopted by global navigation satellite system (GNSS) [4]. However, GNSS signals are often unreliable or even unavailable because they are difficult to be detected by mobile robots all the time in indoor environments [5]. Moreover, the stability and accuracy of GNSS signals are not guaranteed. For these reasons, ultra wideband (UWB) is widely applied for indoor positioning in recent years due to the high resolution, strong anti-jamming performance, low transmit power, and so on [6,7], but it may be affected by multipath effects and the non-line-of-sight (NLOS) in complicated environments, leading to large positioning errors [8]. Unlike the absolute positioning, the relative positioning technology tracks target state incrementally by estimating changes of relative pose. So it is also called dead reckoning (DR) [9]. As a typical application of the relative positioning, inertial navigation system (INS) is usually applied for trajectory tracking of moving objects owing its favorable properties (e.g., high frequency, fast response and high short-term positioning accuracy) [10]. Unfortunately, the positioning errors of INS are accumulated gradually with time and distance, which makes the estimated trajectory deviate from the true one [11]. Thus, INS is not suitable for accurate long-distance indoor navigation and positioning.

With the development of indoor positioning technologies, the INS/UWB integrated positioning system has been extensively studied and widely applied [12,13]. The inertial measurement unit (IMU) is used to obtain the continuous and regular dynamic information. Meanwhile, the UWB location module outputs the discrete positions with noncumulative error [14]. These two heterogeneous data complement and benefit each other in a fusion system, which overcomes the limitation of single sensor in information utilization. However, in practical applications of INS/UWB integrated positioning, UWB data are usually affected by the time-varying noise in complicated indoor environments [15]. Moreover, outliers may appear in measurements. As a result, not only is the optimal estimation lost, but also the divergence of filter occurs [16], which causes large positioning errors especially when measured data is greatly influenced by external environment.

In practical integrated positioning systems, most kinematics/measurement models are complex and nonlinear. Although nonlinear filters such as the extended Kalman filter (EKF) [17,18], unscented Kalman filter (UKF) [19] and cubature Kalman filter (CKF) [20,21] are widely applied to fuse heterogeneous data, their filtering performance may be reduced when there exists the time-varying measurement noise (caused by multipath effects and the NLOS factor) in complicated indoor environments. This is because these filters usually assume the measurement noise is time invariance, which is clearly not in accordance with practical statistics. In view of this, several adaptive filters have been proposed to improve the integrated positioning performance by estimating the statistical characteristics of noise, e.g., innovation adaptive estimation (IAE) and multiple model adaptive estimation (MMAE) [22]. The IAE method may estimate the noise covariance adaptively by analyzing the filter’s innovation sequence and the attenuation factor was introduced [23]. In [24], the fuzzy inference system was used to output the adaptation value of the measurement noise covariance (MNC), which contributes to achieving adaptive estimation for time-varying noise. [25] proposed an enhanced innovation adaptive estimation Kalman filter (EIAE-KF). It can be switched between the prediction model and the update model based on an innovation sequence for getting the optimal estimation. To deal with the influences of unexpected dynamic errors, [26] presented the strong tracking filter (STF) in integrated positioning system. In [27], a simplified Sage–Husa adaptive filter (SHAF) is used to weaken the effect of time-varying noise on filter performance. Although these approaches may improve the integrated positioning performance to some extent, they do not consider the case that outliers occur in measurements.

Thus, [28] established a fuzzy logic system to recognize outliers, and [29] used the chi-square test to judge whether the measured data were outliers or not. Ref. [30] constructed the probability density function (PDF) of different status based on the Kullback–Leibler (KL) divergence theory, and the outlier detection experiments with UWB radar were carried out as well. In [31], based on the orthogonal properties of innovation sequence, an approach was proposed to detect outliers. Thus measurements containing outliers are corrected by using weight coefficients.

Note that above approaches need to assume that the noise covariance changes little or the number of outliers is small. However, these assumptions do not necessarily coincide with the reality, because phenomenons such as wireless signal interference, pedestrians walking and obstacles block may actually exist in complicated indoor environments. These factors make the statistical characteristics of measurement noise change dramatically, but more importantly, more outliers appear in measured data and degrade the performance.

To solve these problems simultaneously, this paper proposes a Sage–Husa fuzzy adaptive filter (SHFAF) for INS/UWB integrated positioning. The error state is modeled for improving the system robustness instead of using the nominal state directly [32]. The IAE method is also considered, and the regulatory factor of the modified innovation contribution weight (ICW) is obtained through fuzzy inference system, so that the MNC can be estimated accurately in real time. Moreover, the sliding window estimation with fading coefficients is introduced to approximate the true innovation covariance. More importantly, it is applied to outliers detection and correction for providing precise positioning results based on the innovation orthogonality.

Compared with existing approaches, this work has the following innovative aspects:

1. An effective framework of INS/UWB positioning system is proposed for autonomous indoor mobile robots in which the proposed modeling approach is simple to implement. This facilitates the design of a Sage–Husa fuzzy adaptive filter and accordingly improves the positioning accuracy and robustness.
2. In our approach, a modified innovation contribution weight is obtained by the fuzzy inference system, which serves our Sage–Husa fuzzy adaptive filter. Thanks to its favorable property (i.e., self-adaptive adjustment), the measurement noise covariance can be estimated accurately.
3. To reduce the adverse effect of outliers in a complicated indoor environment, a sliding window estimation with fading coefficients is presented for outlier detection and correction based on innovation orthogonality criterion.
4. Instead of merely using numerical simulations, we adopt the Gazebo simulation engine to evaluate the positioning performance. The measurement data are generated in real time and complicated factors (e.g., kinematics and interferences) in real scenarios are considered explicitly. More importantly, in this paper, practical experiments are also performed to verify the effectiveness of what we propose.

This paper is organized as follows. Section 2 formulates the INS/UWB integrated positioning problem. Besides, the kinematic and measurement model of INS/UWB are presented, respectively. In Section 3, an effective system framework of INS/UWB integrated positioning is established, where the Sage–Husa fuzzy adaptive filter is easily obtained to improve the positioning performance, the difficult but important problems of time-varying noise and outliers are considered and solved. In Section 4, simulation and experimental results demonstrate the effectiveness of the proposed INS/UWB integrated positioning approach. The last section concludes the paper.

## 2. System Model

### 2.1. Problem Formulation

For the problem of INS/UWB integrated positioning, the key is how to integrate two heterogeneous sensors’ data effectively and reasonably according to the prior information such as kinematic model, measurement model and noise characteristics of moving target, so as to achieve the estimated pose state (i.e., $x^{pose}$) with smaller error than positioning approaches with an individual sensor’s data. It is given by $x^{pose}={\left[p^{T},q^{T}\right]}^{T}$, which consists of the position and attitude (i.e., the orientation of a moving target). This paper focuses on the target pose state estimation in three-dimensional (3D) cartesian coordinates, in which p and q denote position and attitude in 3D space, respectively. Unlike in the 2D space, the pose in 3D space needs more variables to be described. In addition, the attitude vector and the dynamics parameters are nonlinearly coupled (to be demonstrated later), which is difficult to be handled.

To improve the pose estimation performance, other indirect state variables related to the pose are also considered. They are included into the whole state vector in $x_{k}={\left[p_{k}^{T},v_{k}^{T},q_{k}^{T},a_{b,k}^{T},\omega_{b,k}^{T}\right]}^{T}$, where *k* denotes the time index, v, $a_{b}$ and $\omega_{b}$ are the velocity, the offset of acceleration and angular velocity. Consider the following system model:

(1) $$\begin{array}{c} x_{k}=f\left(x_{k-1},u_{k-1},w_{k-1}\right)\\ z_{k}=h\left(x_{k},v_{k}\right) \end{array}$$

It characterizes the target’s kinematics and sensor measurements, where $x_{k}$ is the state vector with transition function f(·), $z_{k}$ is measurement vector obtained by the measurement function h(·). u is the input or control vector, w and v are the process and measurement noise, respectively.

In INS/UWB integrated positioning system, $f(x_{k-1},u_{k-1},w_{k-1})$ is determined by the kinematics of INS, and $h(x_{k},v_{k})$ is obtained by UWB measurement equations. The positioning systems of INS and UWB can be integrated in a unified framework and described by Equation (1). Besides, based on the error state, the kinematic model of INS and UWB measurement model will be established and introduced below.

### 2.2. Kinematic Model

**Figure 1:**
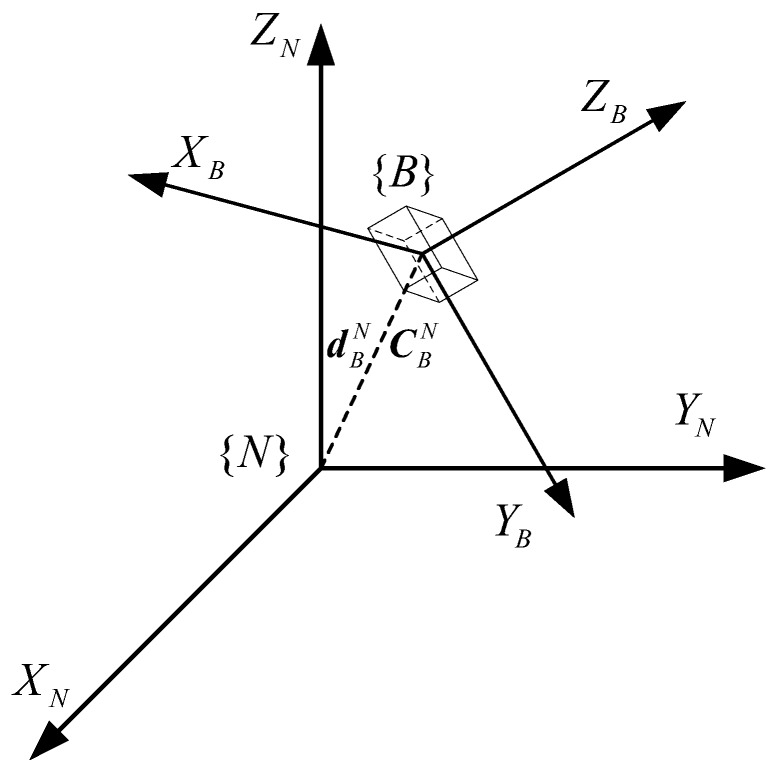
The transformation between the navigation frame and the body frame.

For INS/UWB integrated positioning, the dynamics of a moving target should be analyzed to obtain its state transition equation, which facilitates the kinematic model. Based on the kinematic model, the relative position, velocity and attitude can be incrementally estimated by using the inertial data (i.e., acceleration and angular velocity). In INS, there are two reference frames: body reference frame {*B*} and navigation frame {*N*}. The transformation between these reference coordinates can be shown in Figure 1, in which the transformation from {*B*} to {*N*} is described by the translation vector $d_{B}^{N}$ and the rotation matrix $C_{B}^{N}$ jointly.

Compared with Euler angles, the quaternion may not be affected by the gimbal lock and has one-to-one correspondence with the rotation [33]. Thus it is adopted for attitude representation in state vector. Besides, the rotation matrix is also used in this paper, which can be transformed by the quaternion with the following equation [34]:

(2) $$C=\left(2{q_{0}}^{2}-1\right)I_{3}-2q_{0}{\left[q_{v}\right]}_{\times }+2q_{v}q_{v}^{T},$$

where ${\left[\cdot \right]}_{\times }$ is the skew symmetric operator, $q_{0}$ and $q_{v}={\left[q_{1},q_{2},q_{3}\right]}^{T}$ are the real and imaginary part of the rotation quaternion, respectively.

The joint state vector of INS/UWB positioning system in the navigation coordinates {*N*} is $x={\left[p^{T},v^{T},q^{T},a_{b}^{T},\omega_{b}^{T}\right]}^{T}$. It consists of the nominal state $\hat{x}$ and the error state δx, which satisfies

(3) $$x=\hat{x}⊕\delta x,$$

where $\delta x={\left[\delta p^{T},\delta v^{T},\delta \theta^{T},\delta a_{b}^{T},\delta \omega_{b}^{T}\right]}^{T}$. ⊕ denotes a generic composition (quaternion products for the quaternion states and sums for other states). Note that δθ is the angle error vector respecting to the three axes of body coordinates {*B*}. Since δθ is very small, the rotation error of quaternion can be approximately expressed as $\delta q={\left[1,\delta \theta^{T}/2\right]}^{T}$. Correspondingly, the transition of the nominal state and error state has the following forms [32]:

(4) $$\dot{\hat{x}}=\left[\begin{array}{c} \dot{\hat{p}}\\ \dot{\hat{v}}\\ \dot{\hat{q}}\\ {\dot{\hat{a}}}_{b}\\ {\dot{\hat{\omega }}}_{b} \end{array}\right]=\left[\begin{array}{c} \hat{v}\\ \hat{C}\left(a_{m}-{\hat{a}}_{b}\right)+g\\ \frac{1}{2}\hat{q}⊗\left(\omega_{m}-{\hat{\omega }}_{b}\right)\\ 0\\ 0 \end{array}\right]$$

(5) $$\delta \dot{x}=\left[\begin{array}{c} \delta \dot{p}\\ \delta \dot{v}\\ \delta \dot{\theta }\\ \delta {\dot{a}}_{b}\\ \delta {\dot{\omega }}_{b} \end{array}\right]=\left[\begin{array}{c} \delta v\\ -\hat{C}{\left[a_{m}-{\hat{a}}_{b}\right]}_{\times }\delta \theta -\hat{C}\delta a_{b}-\hat{C}a_{n}\\ -{\left[\omega_{m}-{\hat{\omega }}_{b}\right]}_{\times }\delta \theta -\omega_{w}-\omega_{n}\\ a_{w}\\ \omega_{w} \end{array}\right],$$

where g is the gravity acceleration vector in {*N*}, $a_{m}$ and $\omega_{m}$ are the acceleration and angular velocity measurements, respectively. $a_{n}$ and $\omega_{n}$ are the measurement noise of the accelerometer and gyroscope, respectively. $a_{w}$ and $\omega_{w}$ are the random walk noise of biases.

### 2.3. Measurement Model

Generally, the processed UWB measurements provide an absolute reference for the integrated positioning system and are used to correct the accumulative error of INS, which are modeled to the position of UWB tag. To improve the positioning accuracy, several UWB anchors may be used sometimes. However, overdetermined equations also may appear in the process of dealing with range measurements obtained by using time of arrival (TOA). So in this paper, the least squares (LS) method was used for more accurate position estimation of UWB tag, which is demonstrated as follows.

Assuming that the position vector of UWB anchors in navigation coordinates is $p_{n}={\left[x_{n},y_{n},z_{n}\right]}^{T}$, where n∈{1,2,3,⋯,N} denotes the anchor’s serial number, and *N* is the total number of anchors. If the position vector of the UWB tag at time *k* is $p_{m,k}={\left[x_{m,k},y_{m,k},z_{m,k}\right]}^{T}$, then the measurement distance from the UWB tag to the *n*-th anchor is written as

(6) $$d_{n,k}={∥p_{n}-p_{m,k}∥}_{2}=\sqrt{{\left(x_{n}-x_{m,k}\right)}^{2}+{\left(y_{n}-y_{m,k}\right)}^{2}+{\left(z_{n}-z_{m,k}\right)}^{2}}.$$

Similarly, if the true position of tag is $p_{t,k}$, the true distance can be defined as

(7) $${\bar{d}}_{n,k}={∥p_{n}-p_{t,k}∥}_{2}=\sqrt{{\left(x_{n}-x_{t,k}\right)}^{2}+{\left(y_{n}-y_{t,k}\right)}^{2}+{\left(z_{n}-z_{t,k}\right)}^{2}}.$$

The positioning problem of the tag can be formulated as finding the solution ${\hat{p}}_{m,k}$ which minimizes the the error between the measurement distance and true distance, i.e.,

(8) $${\hat{p}}_{m,k}=\underset{p_{m,k}}{\arg\min}\sum_{n=1}^{N}{\left(d_{n,k}-{\bar{d}}_{n,k}\right)}^{2}$$

Note that *N* may be greater than the dimension of the unknown position vector. From Equation (6), we have

(9) $$\left\{\begin{array}{c} d_{1,k}^{2}={\left(x_{1}-x_{m,k}\right)}^{2}+{\left(y_{1}-y_{m,k}\right)}^{2}+{\left(z_{1}-z_{m,k}\right)}^{2}\\ d_{2,k}^{2}={\left(x_{2}-x_{m,k}\right)}^{2}+{\left(y_{2}-y_{m,k}\right)}^{2}+{\left(z_{2}-z_{m,k}\right)}^{2}\\ \vdots \\ d_{N,k}^{2}={\left(x_{N}-x_{m,k}\right)}^{2}+{\left(y_{N}-y_{m,k}\right)}^{2}+{\left(z_{N}-z_{m,k}\right)}^{2}. \end{array}\right.$$

Let the second row, third row, …, and *N*-th row subtract the first row, respectively. A linear equation is obtained as

(10) $$2Gp_{m,k}=b_{k},$$

where

(11) $$G=\left(\begin{array}{ccc} x_{1}-x_{2} & y_{1}-y_{2} & z_{1}-z_{2}\\ \vdots & \vdots & \vdots \\ x_{1}-x_{N} & y_{1}-y_{N} & z_{1}-z_{N} \end{array}\right)$$

(12) $$b_{k}=\left(\begin{array}{c} d_{2,k}^{2}-d_{1,k}^{2}+x_{1}^{2}-x_{2}^{2}+y_{1}^{2}-y_{2}^{2}+z_{1}^{2}-z_{2}^{2}\\ \vdots \\ d_{N,k}^{2}-d_{1,k}^{2}+x_{1}^{2}-x_{N}^{2}+y_{1}^{2}-y_{N}^{2}+z_{1}^{2}-z_{N}^{2}. \end{array}\right)$$

Accordingly, Equation (10) can be solved by using the least squares method:

(13) $$p_{m,k}=\frac{1}{2}{\left(G^{T}G\right)}^{-1}G^{T}b_{k}.$$

Meanwhile, the velocity of UWB tag is also obtained as

(14) $$v_{m,k}=\frac{\Delta p_{k}}{\Delta t_{k}}=\frac{p_{m,k}-p_{m,k-1}}{t_{k}-t_{k-1}}.$$

Suppose that the position and velocity vector of INS at time *k* are $p_{INS,k}$ and $v_{INS,k}$ respectively, then the measurement equation of the integrated positioning system is

(15) $$z_{k}=\left[\begin{array}{c} \delta p_{k}\\ \delta v_{k} \end{array}\right]=\left[\begin{array}{c} p_{m,k}-p_{INS,k}\\ v_{m,k}-v_{INS,k} \end{array}\right]=H\delta x_{k}+v_{k},$$

where v∼N{0,R} is the measurement noise vector, R is its covariance matrix, and $H=\left[\begin{array}{ccccc} I_{3} & 0 & 0 & 0 & 0\\ 0 & I_{3} & 0 & 0 & 0 \end{array}\right]$ is the measurement matrix of the error state.

## 3. INS/UWB Integration Strategy with Sage–Husa Fuzzy Adaptive Filter

**Figure 2:**
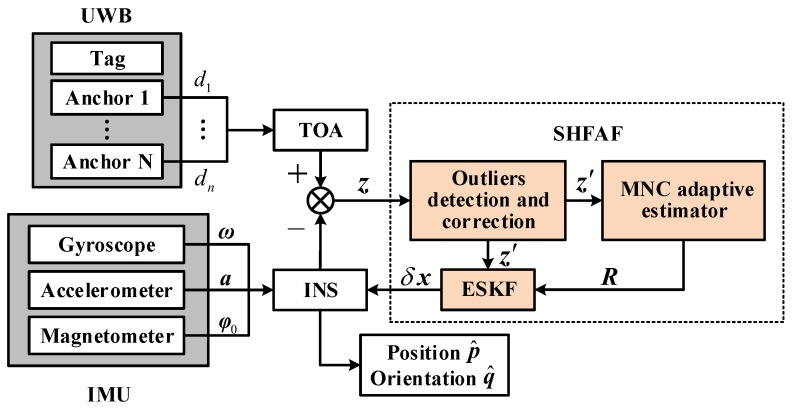
The framework of robust inertial navigation systems (INS)/ultra wideband (UWB) integrated positioning system.

To achieve precise positions of moving targets using two different types of data (measured from IMU and UWB), this paper proposes a framework of INS/UWB integrated positioning system. As shown in the framework of Figure 2, ω, α, $\varphi_{0}$ are measured by IMU, which serve as the inputs of INS. Thus the position, velocity and attitude of a robot can be obtained by using recursive integration, in which the measurements of triaxial magnetometer are used for its orientation initialization. Absolute measurements (i.e., position and velocity) in navigation coordinates are obtained from UWB positioning system by using the TOA method based on the distances between UWB anchors and tag. In this INS/UWB integrated positioning system, UWB measurements are used to correct the INS output. However, since indoor environments are often complicated (dynamic, occlusion factors, etc.), the UWB measurements are usually affected by the multipath effects and NLOS factor. This may bring the outliers and therefore cause the performance degradation or even filter divergence.

In view of the above, this paper proposes a Sage–Husa fuzzy adaptive filter (SHFAF) based on the error state. It has a favorable property that the nonlinear nominal state estimation problem can be transformed into a optimal linear one [35]. More importantly, the estimation accuracy and robustness improved with low computational complexity.

As shown in Figure 2, SHFAF included the following modules: outlier detection and correction, MNC adaptive estimator, and error state Kalman filter. The module of outliers detection and correction aims to recognize outliers and reduce the deviation degree of them by pre-processing the raw measurements. In MNC adaptive estimator, the time-varying MNC can be estimated accurately based on the innovation adaptive estimation. Moreover, the corrected measurements and their estimated noise covariance are as the inputs of error state Kalman filter. Finally, the compensation values calculated by SHFAF were injected into the outputs of INS, and the pose estimation of robot can be achieved. The implementation of proposed SHFAF is explained in detail below.

### 3.1. Prediction

From Equation (3), the whole state vector is composed of the nominal state and error state, which can be predicted by the transition equation recursively. The nominal state prediction is written as

(16) $${\hat{x}}_{k|k-1}=f\left({\hat{x}}_{k-1},u_{k-1}\right),$$

where f(·) denotes the nonlinear state transition function. It can be derived by the Euler integrals of Equation (4). As the control inputs, $u_{k}={\left[a_{m,k}^{T},\omega_{m,k}^{T}\right]}^{T}$ are measurements of the triaxial accelerometer and gyroscope. The process noise in error state transition is accumulated over time, and the uncertainty of error state can be reflected by a covariance matrix. Correspondingly, the prediction of the error state and its covariance are

(17) $$\delta x_{k|k-1}=F_{\delta x,k-1}\left({\hat{x}}_{k-1},u_{k-1}\right)\cdot \delta x_{k-1}$$

(18) $$P_{k|k-1}=F_{\delta x,k-1}P_{k-1}F_{\delta x,k-1}^{T}+\Gamma_{n}Q_{n}\Gamma_{n}^{T}.$$

Note that since the mean of the error state δx is initialized to zero, the linear Equation (17) always returns zero. $F_{\delta x,k-1}$ is the error state transition matrix, it can be obtained by the discretization of Equation (5):

(19) $$F_{\delta x,k-1}=\left[\begin{array}{ccccc} I_{3} & I_{3}\Delta t & 0 & 0 & 0\\ 0 & I_{3} & -{\hat{C}}_{k-1}{\left[a_{m,k-1}-{\hat{a}}_{b,k-1}\right]}_{\times }\Delta t & -{\hat{C}}_{k-1}\Delta t & 0\\ 0 & 0 & {\hat{C}}_{k-1}\left\{\left(\omega_{m,k-1}-{\hat{\omega }}_{b,k-1}\right)\Delta t\right\} & 0 & -I_{3}\Delta t\\ 0 & 0 & 0 & I_{3} & 0\\ 0 & 0 & 0 & 0 & I_{3} \end{array}\right]$$

(20) $$\Gamma_{n}=\left[\begin{array}{cccc} 0 & 0 & 0 & 0\\ I_{3} & 0 & 0 & 0\\ 0 & I_{3} & 0 & 0\\ 0 & 0 & I_{3} & 0\\ 0 & 0 & 0 & I_{3} \end{array}\right]$$

(21) $$Q_{n}=\left[\begin{array}{cccc} {\left(\sigma_{a_{n}}\Delta t\right)}^{2}I_{3} & 0 & 0 & 0\\ 0 & {\left(\sigma_{\omega_{n}}\Delta t\right)}^{2}I_{3} & 0 & 0\\ 0 & 0 & {\left(\sigma_{a_{\omega }}\Delta t\right)}^{2}I_{3} & 0\\ 0 & 0 & 0 & {\left(\sigma_{\omega_{\omega }}\Delta t\right)}^{2}I_{3} \end{array}\right],$$

where $\Gamma_{n}$ is the noise driven matrix and $Q_{n}$ is the process noise covariance matrix.

### 3.2. Covariance Adaptive Estimator of Measurement Noise

The adaptive Kalman filter is usually applied in complex and unknown noise circumstances, because it estimates the noise covariance at each recursive step. In the traditional Kalman filter framework, however, the noise covariance is a constant matrix. Thus the innovation $\varepsilon_{k}$ is defined as

(22) $$\varepsilon_{k}=z_{k}-H\delta x_{k|k-1},$$

and its theoretical covariance is

(23) $$S_{k}=HP_{k|k-1}H^{T}+R_{k}.$$

The theoretical covariance equals the truth when the filter is strictly converged. Correspondingly, the convergence criterion is derived as

(24) $$\varepsilon_{k}\varepsilon_{k}^{T}=HP_{k|k-1}H^{T}+R_{k},$$

and the theoretical estimate of MNC matrix ${\hat{R}}_{k}$ is

(25) $${\hat{R}}_{k}=\varepsilon_{k}\varepsilon_{k}^{T}-HP_{k|k-1}H^{T}.$$

When the filter runs, the MNC matrix is estimated recursively, and smooth operation is necessary to ensure its continuity. To synthesize the above analysis, the Sage–Husa fuzzy adaptive estimator can be represented by

(26) $$R_{k}=\left(1-s_{k}^{\alpha }d_{k}\right)R_{k-1}+s_{k}^{\alpha }d_{k}\left(\varepsilon_{k}\varepsilon_{k}^{T}-HP_{k|k-1}H^{T}\right).$$

In this equation, $d_{k}$ is the modified innovation contribution weight (MICW)

(27) $$d_{k}=\left(\lambda -b\right)/\left(\lambda -b^{k+1}\right),$$

where λ≥1. $d_{k}$ reduces to the traditional ICW when λ=1. The value λ is used to increase the asymptotic stability value of ICW which provides a wide range for the output of the fuzzy inference system, *b* is a forgetting factor (usually between 0.95 and 0.99), and $s_{k}$ is a regulatory factor of MICW which can be obtained by the fuzzy inference system.

**Remark** **1.** *The estimated MNC at time k (i.e., $R_{k}$) is weighted by two parts; one is the covariance of previous sampling time (i.e., $R_{k-1}$), the other part is the difference between the estimated and theoretical covariance of innovation at time k. Since the traditional ICW is very small after stabilization, the information provided by innovation is little as well. When time-varying noise is observed, the changes of MNC may not be estimated in time. Therefore, the traditional ICW is modified as Equation (27), and it will be analyzed in detail later*.

In this paper, we have made two improvements to ICW; (a) λ in Equation (27) was introduced to increase the steady-state value of ICW, and the contribution of innovation to $R_{k}$ was increased, and (b) the regulatory factor (i.e., $s_{k}$) of MICW was obtained through fuzzy inference for improving the estimation ability to $R_{k}$.

**Figure 3:**
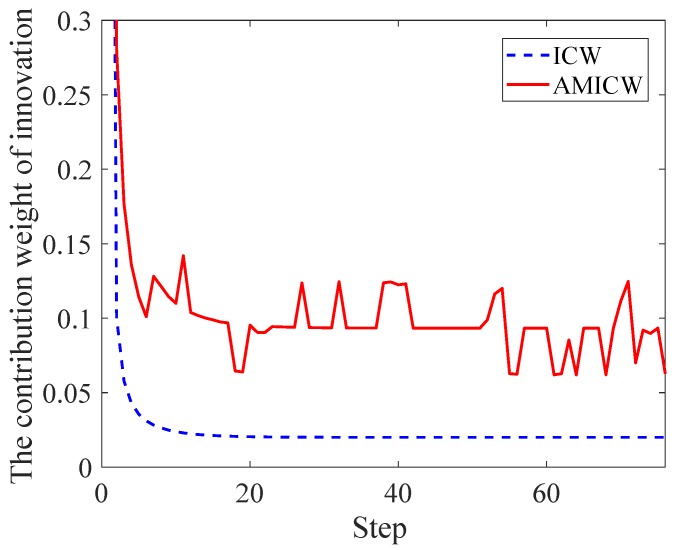
The changes of innovation contribution weight (ICW) and adaptive modified innovation contribution weight (AMICW).

We define the adaptive modified innovation contribution weight (AMICW) as the product of regulatory factor and MICW (i.e., $s_{k}d_{k}$), then the changes of ICW and AMICW are compared in Figure 3. It can be seen that AMICW always maintained a larger scale and can be adjusted adaptively according to the changes of external noise characteristics. In comparison, the value of ICW was very small and basically invariant after several iterations, which made little contribution to the estimate of $R_{k}$.

To obtain the regulatory factor $s_{k}$, we construct a fuzzy reference system. As the input of a fuzzy reference system, the difference coefficient $r_{k}$ between the estimated innovation covariance ${\hat{S}}_{k}$ and the theoretical covariance $S_{k}$ should be calculated first, i.e.,

(28) $${\hat{S}}_{k}=E\left(\varepsilon_{k}\varepsilon_{k}^{T}\right)$$

(29) $$r_{k}=\left|\frac{\mathrm{Tr}({\hat{S}}_{k})}{\mathrm{Tr}(S_{k})}-1\right|,$$

where $S_{k}$ is calculated through Equation (23), and Tr(·) is the trace of a matrix. Then, the difference coefficient $r_{k}$ serves as the input of the fuzzy reference system, and the corresponding output $s_{k}$ can be obtained:

(30) $$s_{k}=\left\{\begin{array}{cc} 1\;, & k\leq k_{s}\\ fuzzy(r_{k}), & k>k_{s}, \end{array}\right.$$

where fuzzy(·) is regarded as a nonlinear function. Because of the unknown a priori information of measurement noise, an initial estimate of R is obtained by the simplified Sage–Husa adaptive filter at the beginning (i.e., $k\leq k_{s}$). When $k>k_{t}$, MICW tends to be stable. At this stage, the regulatory factor $s_{k}$ was inferred by the fuzzy inference system and plays a dominant role in adaptive tuning.

**Remark** **2.** *The first adaption for MICW relies on the forgetting factor b, and it is adjusted adaptively in the second time through the regulatory factor $s_{k}$. As a scaling of $s_{k}$, α is chosen depending on the practical situations. A larger α approaches a true R by using fewer iterations, but this way leads to unstable estimates (e.g., the estimation oscillates around the ground truth); a smaller α may smooth the estimator, while more steps are necessary to reach the ground truth*.

The inference rules of the above fuzzy logic system are written as

(31) $$\begin{array}{cc} If & r_{k}\in \mathrm{Equal},\;then\;s_{k}\in \mathrm{Equal}\\ If & r_{k}\in \mathrm{More}\;,\;then\;s_{k}\in \mathrm{More}\\ If & r_{k}\in \mathrm{Less}\;\;,\;then\;s_{k}\in \mathrm{Less} \end{array}$$

**Figure 4:**
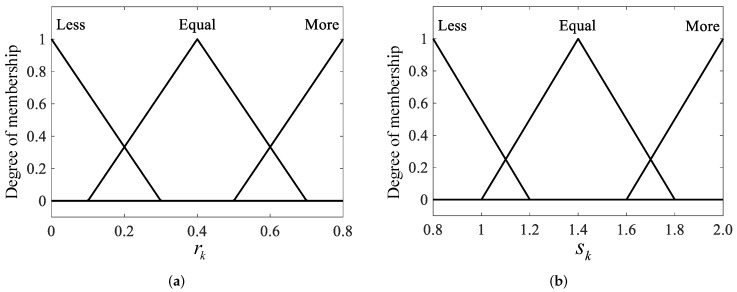
The membership function of input and output. (**a**) The input of fuzzy inference system. (**b**) The output of fuzzy inference system.

According to Equation (29), it can be seen that $r_{k}$ intuitively reflects the error between the estimated and theoretical value of innovation covariance, which characterizes the change degree of MNC. The larger the $r_{k}$ is, the greater the error between the estimated and theoretical value is. This means the external noise statistical characteristics have changed a lot, and correspondingly more extra information is required to correct $R_{k}$. Conversely, the smaller the $r_{k}$, the smaller the contribution of innovation to the estimated MNC. In this case, the present $R_{k}$ should be estimated by more information of the previous $R_{k}$ (i.e., slightly decreasing $s_{k}$). Thus $s_{k}$ should be increased to enhance the innovation contribution for the estimate of $R_{k}$. The input and output membership function of fuzzy inference system are shown in Figure 4.

Through two stages of adaptive estimation, the MNC can be accurately estimated in the whole process, and the performance of the filter can be improved.

### 3.3. Outlier Detection and Correction

UWB may affected by unknown and uncertain disturbances such as multipath effects and NLOS factor. In this case, the outliers exist in measurements and are far away from the ground truth. This phenomenon whose adverse impact on the filter performance is difficult to be reduced only by IAE method. To avoid large estimation error and even the filter divergence, this paper proposes approach to the outliers detection and correction based on the innovation orthogonal criterion. According to the orthogonality of innovation, we have

(32) $$E(z_{k}z_{k}^{T})=E(\varepsilon_{k}\varepsilon_{k}^{T})+E\left(z_{k|k-1}z_{k|k-1}^{T}\right),$$

and the theoretical expectation of $z_{k}z_{k}^{T}$ is

(33) $$D_{k}=HP_{k|k-1}H^{T}+R_{k}+H\delta x_{k|k-1}\delta x_{k|k-1}^{T}H^{T}.$$

It can be judged whether the measurement at time *k* is an outlier or not by calculating the ratio between the estimated and theoretical expectation of $z_{k}z_{k}^{T}$. Suppose that the *i*-th diagonal element of $E(z_{k}z_{k}^{T})$ and $D_{k}$ are $G_{i,k}$ and $D_{i,k}$, respectively. If $M_{i,k}=G_{i,k}/D_{i,k}$, then

(34) $$fr_{i,k}=\left\{\begin{array}{c} 1\\ 1/\sqrt{M_{i,k}} \end{array}\right.\;\begin{array}{c} M_{i,k}\leq \xi_{i}\\ M_{i,k}>\xi_{i}, \end{array}$$

where $\xi_{i}$ is the predetermined sensitivity threshold of outliers recognition. Thus the outlier is corrected as

(35) $$z_{k}^{\prime }=fr_{k}\times z_{k},$$

where $fr_{k}=diag\left[fr_{1,k},fr_{2,k},\cdots ,fr_{rank(R),k}\right]$. From Equation (34) we can see that the larger $M_{i,k}$ (i.e., the deviation between measurement and prediction) is, the smaller $fr_{i,k}$.

To approximate the true $E(z_{k}z_{k}^{T})$ in Equation (32), the estimated innovation covariance ${\hat{S}}_{k}$ and the expectation $E(z_{k|k-1}z_{k|k-1}^{T})$ need to be calculated. The value ${\hat{S}}_{k}$ is usually generated by sliding window estimation, in which the innovation sequence consisting of the recent *N* innovations is used to approximate the true covariance at the current time. However, the statistical characteristics of innovations obtained by the traditional sliding window estimation may only reflect their average status. Once outliers appear, the covariance may not vary greatly (especially when the size of sliding window is large), causing the outliers not to be captured correctly and timely.

**Remark** **3.** *Reducing the width of sliding window can enhance the tracking sensitivity to the innovation covariance, but the estimate accuracy of innovation covariance may be degraded if the window is getting too narrow*.

To improve the ability to capture outliers, the coefficient series with the ‘forgotten’ properties are weighted to the innovation sequence. This makes new data play a major role (i.e., given by a larger weight), and the innovations far away from filtering moment are conferred smaller weights. As a result, the sensitivity to outliers can be improved while maintaining the width of the sliding window unchanged. The sliding window estimation is modified as

(36) $${\hat{S}}_{k}=E\left(\varepsilon_{k}\varepsilon_{k}^{T}\right)=\sum_{j=k-l+1}^{k}\sigma_{j}\varepsilon_{j}\varepsilon_{j}^{T},$$

where $\sigma_{j}=a^{k-j}\left(1-a\right)/\left(1-a^{l}\right)$ is the fading coefficient of the *j*-th innovation covariance in a sliding window with the width of *l*, and *a* characterizes the fading rate (usually taken to be between 0.95 to 0.99).

### 3.4. Measurement Update

After the above steps, the Kalman gain K of the error state $\delta x_{k}$ can be calculated, i.e.,

(37) $$K_{k}=P_{k|k-1}H^{T}{\left(HP_{k|k-1}H^{T}+R_{k}\right)}^{-1}.$$

The estimated error state vector and its corresponding covariance are updated as

(38) $$\delta x_{k}=K_{k}\varepsilon_{k}$$

(39) $$P_{k}=\left(I-K_{k}H\right)P_{k|k-1}.$$

Thus the error state is injected into the nominal state, and the true state is estimated by Equation (3). Particularly,

(40) $$\left[\begin{array}{c} {\hat{p}}_{k}\\ {\hat{v}}_{k}\\ {\hat{q}}_{k}\\ {\hat{a}}_{b,k}\\ {\hat{\omega }}_{b,k} \end{array}\right]=\left[\begin{array}{c} {\hat{p}}_{k-1}+\delta p_{k}\\ {\hat{v}}_{k-1}+\delta v_{k}\\ {\hat{q}}_{k-1}⊗q\left\{\delta \theta_{k}\right\}\\ {\hat{a}}_{b,k-1}+\delta a_{b,k}\\ {\hat{\omega }}_{b,k-1}+\delta \omega_{b,k} \end{array}\right].$$

**Figure 5:**
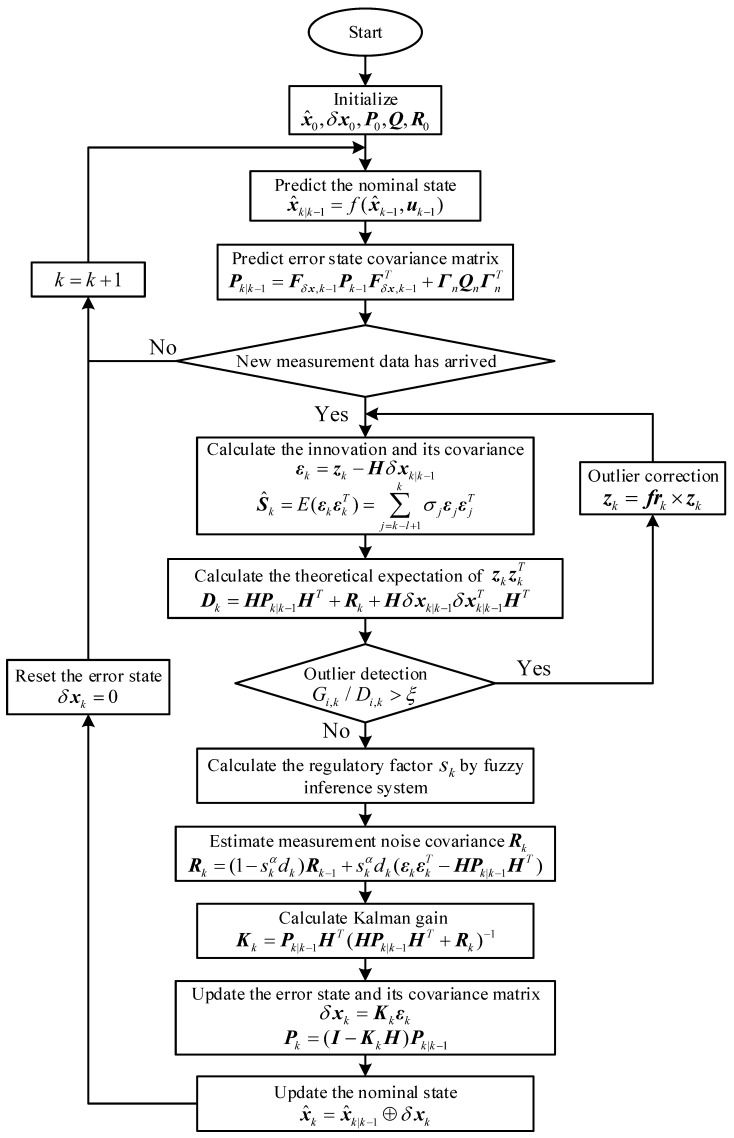
The flowchart of Sage–Husa fuzzy adaptive error state Kalman filter.

Afterwards, the estimator (i.e., ${\hat{x}}_{k}$) is considered as the nominal state for next recursion. Note that the estimate of the error state should be reset to zero after nominal states have been updated. The flow chart of our algorithm is shown in Figure 5.

## 4. Experiment Analysis and Evaluation

In this section, the proposed INS/UWB integrated positioning approach is evaluated and analyzed to illustrate its effectiveness; a) two simulation scenarios (only the time-varying noise and the time-varying noise with outliers in measurements) are provided to test the convergence and robustness of our approach and b) the experimental platform of INS/UWB integrated positioning system is established, where the practical positioning experiments in indoor environments with two different motion trajectories of a mobile robot are performed to verify the effectiveness of the proposed approach in real applications.

Note that several different positioning approaches are compared in our simulations and practical experiments, including TOA based UWB positioning (see Section 2.3), ESKF [32], SHAF [36] and SHFAF.

### 4.1. Simulation Experiments and Analysis

**Figure 6:**
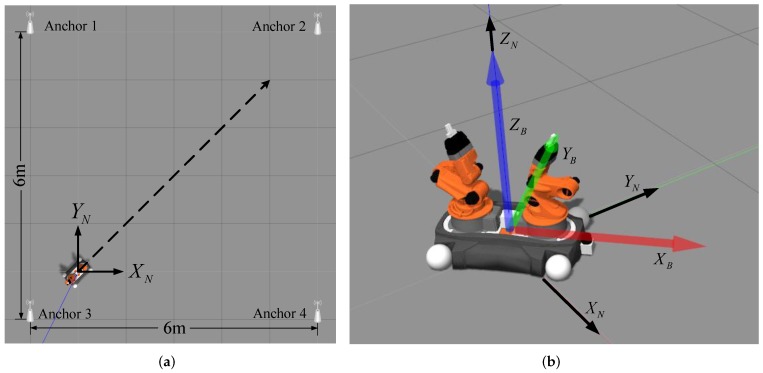
The simulation experiment in Gazebo. (**a**) The initial pose, desired trajectory of mobile robot and distribution of anchors. (**b**) The navigation and body coordinates in simulations.

In this part, simulations are performed by the Gazebo physical simulation engine. The east–north–up coordinate system is adopted for our navigation, and four UWB anchors are used and located in (−5,−1), (1,−1), (1,5), (−5,5) (see Figure 6a). UWB tag is fixed on the geometric center of a robot body. The UWB measurement noises are generally assumed to consist of the ranging noise and the time-varying noise [37]. The later is usually caused by NLOS scenarios, multipath effects and other uncertain factors. The ranging noise is zero-mean Gaussian white noise and its variance is $0.1^{2}$. The interference noise is random with uniform distributed (i.e., u(−0.2,0.2)). The update frequency of UWB data is 5 Hz, and the TOA-based positioning is used to get the tag position. The IMU and UWB tag are placed in the same location for collecting the acceleration and angular velocity of the robot in simulation environments. The update frequency of IMU data is 100 Hz, and Gaussian noise parameter is $0.06^{2}$.

In simulations, the initial position of this robot is located at (0,0) in the navigation coordinates, it moves along the diagonal line of 4 m × 4 m square with constant speed 0.4 m/s. Note that there is a relative transformation between the initial body coordinates and the navigation coordinates, which can be seen in Figure 6b. As the robot moving on, IMU and UWB measurements are recorded and processed simultaneously. Meanwhile, several different positioning approaches are compared for performance evaluation.

To verify the ability of self-adaptation and outliers suppression of the proposed INS/UWB integrated positioning approach, two different scenarios are designed as follows.

**Scenario 1**: The measurement noises are composed of the Gaussian white noise and random walk noise (without outliers). The aim of this experiment is to test the convergence and adaptive capacity of the presented approach. The filter is initialized at (0.57,0), i.e., it starts from a larger error point due to lack of information. In addition, the MNC is initialized with a large matrix, which means that there exists great uncertainty in the initial stage.

**Figure 7:**
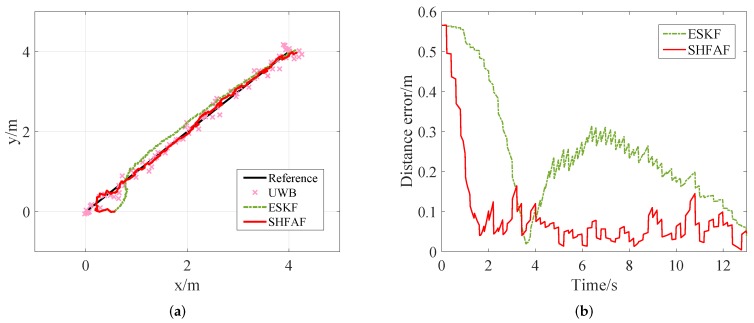
The simulation results in Scenario 1. (**a**) The estimated position trajectory in which outliers are not considered, but time-varying noise and initial error are set up. (**b**) The absolute distance error of positioning results.

Simulation results for this scenario are show in Figure 7, where the absolute error is chosen as a measurable indicator for position accuracy. Figure 7a gives the estimated trajectories without considering outliers. Clearly, SHFAF outperforms ESKF because it has a stronger adaptive ability to the time-varying noise and its performance is not affected by the initial condition of MNC. This leads to poor positioning accuracy, and even worse than the results of UWB-only positioning at the initial stage. From Figure 7b we can see that the absolute positioning error of SHFAF is less than 0.2 m after 2 s, while ESKF needs about 10 s to achieve such an effect. It can be concluded that SHFAF has faster convergence speed and better self-adaptive ability than ESKF.

**Scenario 2**: Unlike in Scenario 1, outliers exist in measurements with Gaussian white noise and random walk noise. This scenario is designed to evaluate outlier suppression and adaptive ability of the proposed approach comprehensively.

**Figure 8:**
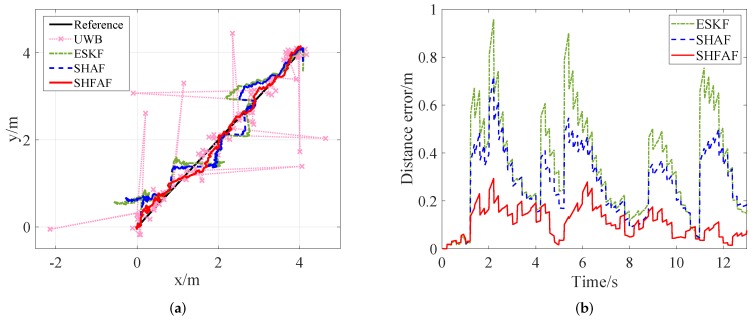
The simulation results in Scenario 2. (**a**) The positioning results that outliers with large errors exist in measurement data. (**b**) The absolute distance error of different integrated positioning approaches.

Figure 8 shows that the position is not estimated accurately only by using UWB when outliers exist. Meanwhile, the positioning error can be reduced effectively by using INS/UWB integrated approach. That is, the position estimation of SHFAF is little affected because the outliers processing and MNC adaptive estimator are implemented together. SHFAF has good accuracy and its positioning errors are mainly below 0.2 m. Conversely, ESKF causes large errors when meeting outliers because it lacks of the ability of adaptive estimation to MNC. Compared with ESKF, SHAF has the self-adaptive ability to the time-varying noise so that smaller positioning error can be achieved. Though SHAF outperforms ESKF, it is still difficult to apply it in complicated environments because of low positioning accuracy.

To evaluate the performance of the proposed SHFAF from a quantitative view, the root mean square error (RMSE) of robot positions in the east, north and absolute distance are calculated, which are listed in Table 1. Clearly, SHFAF has the lowest positioning error among all four approaches in this table. Simulation results demonstrate the benefit of the proposed positioning approach—SHFAF has strong robustness and good self-adaptive ability.

### 4.2. Experimental Analysis and Performance Evaluation

**Figure 9:**
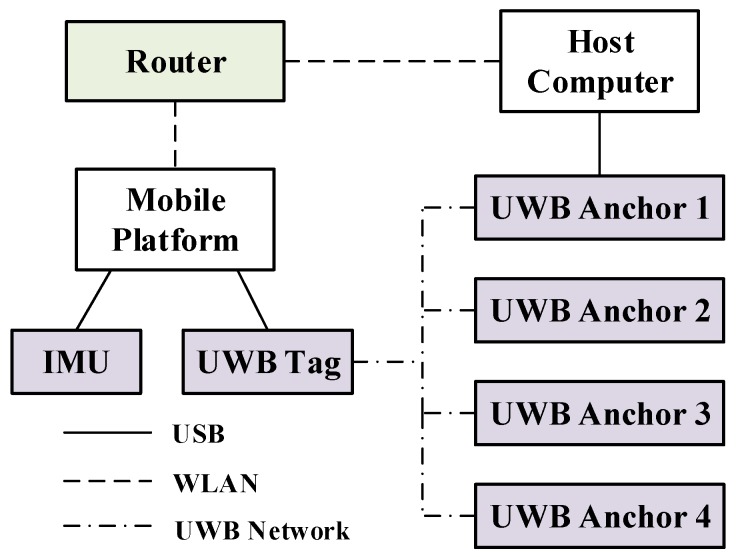
The hardware communication of INS/UWB integrated system.

To further evaluate the performance of the proposed positioning approach in real scenarios, we build a INS/UWB integrated positioning platform (see Figure 9), and the technical specifications of sensors are listed in Table 2. Note that the inertial data are collected by 9-DOF Razor IMU of Sparkfun. The IMU is integrated with a three-axis gyroscope, accelerometer and magnetometer, which serves to measure the nine-DOF inertial information. Besides, this paper used the TREK1000 indoor positioning suite of Decawave based on the DW1000 chip. It consists of four UWB anchors and one UWB tag. The 3D position of a tag can be solved by this system which is considered as an independent positioning system. Communications between UWB modules are built on channel 2 in the frequency range from 3.74 GHz to 4.24 GHz, while the router works on the 2.4 GHz frequency band. So in this case, these two wireless networks hardly interfere with each other.

**Figure 10:**
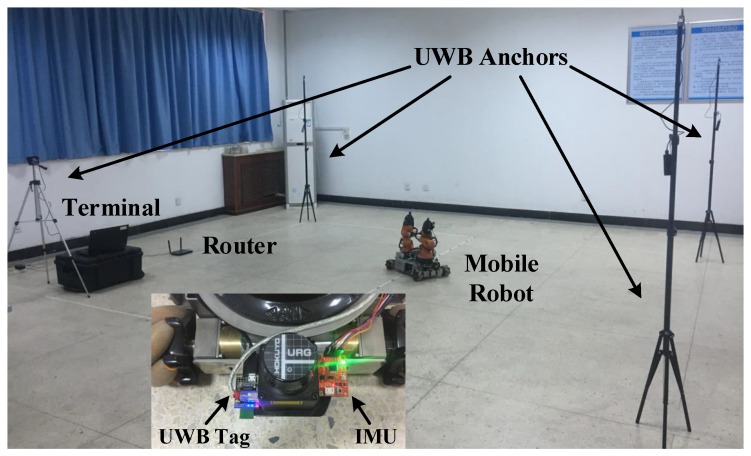
The INS/UWB integrated positioning experimental platform.

A large experimental site is obviously beneficial, so we make the robot cover space as large as possible under the limited experimental conditions. The configuration of the experimental site refers to [11,38], which is shown in Figure 10. The navigation coordinates is established in a rectangular area (i.e., 6.4 m × 4.8 m) which is surrounded by four UWB anchors. They are located at (0,−0.5), (6.4,0.5), (0,4.3) and (6.4,4.3), respectively. All anchors and tag work in the same frequency band and communicate with each other. The sampling frequency of IMU data is 50 Hz, and the update frequency of UWB positioning data is 5 Hz.

**Figure 11:**
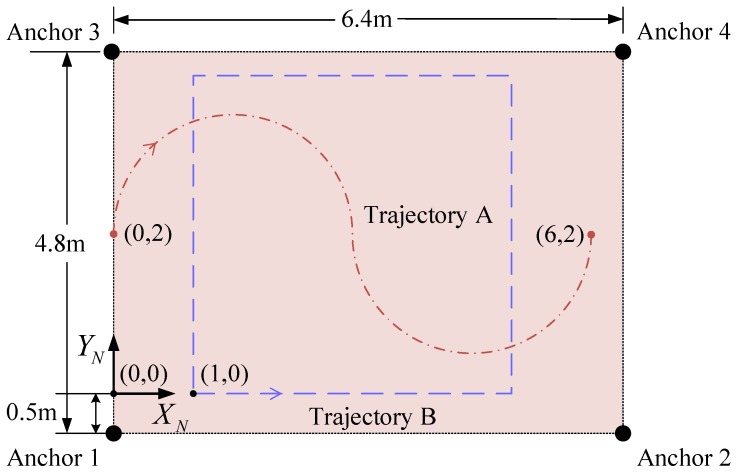
The reference trajectory of integrated positioning experiments.

In a practical experiment, we designed two different motion trajectories to test our positioning approach (see Figure 11). In trajectory A, a robot started from point (0,2) and headed to the north. This trajectory was an S-shaped curve consisting of two semicircles with 1.5 m radius. The linear velocity was 0.405 m/s, and the angular velocity of the two semicircular trajectories was −0.27 rad/s and 0.27 rad/s, respectively. Finally, the robot stopped at point (6,2). Unlike trajectory A, trajectory B was a 4 m × 4 m square in which a robot moved around the edge of the square counterclockwise, i.e., it started at (1,0) and passed through (5,0), (5,4), and (1,4). Finally, this robot returned to the starting point.

**Figure 12:**
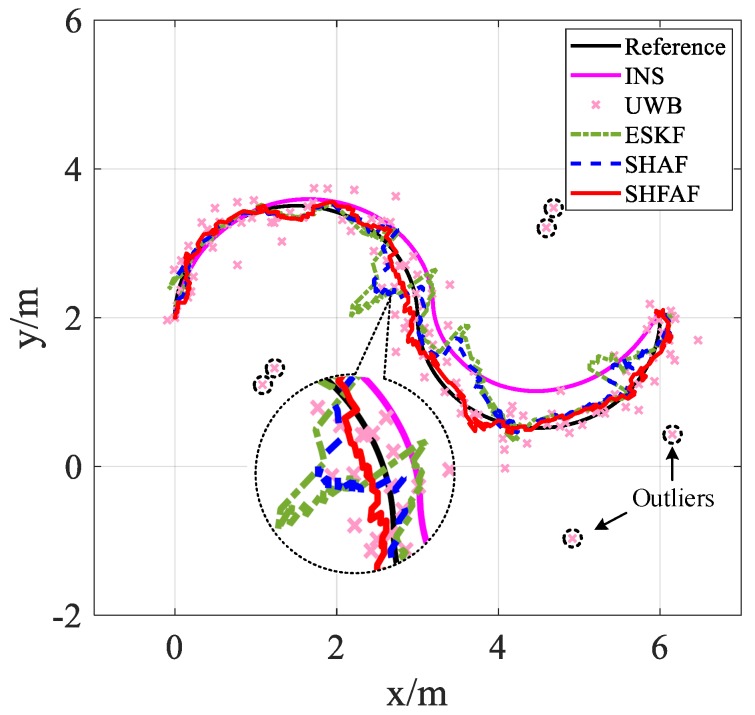
The positioning results of Trajectory A.

**Figure 13:**
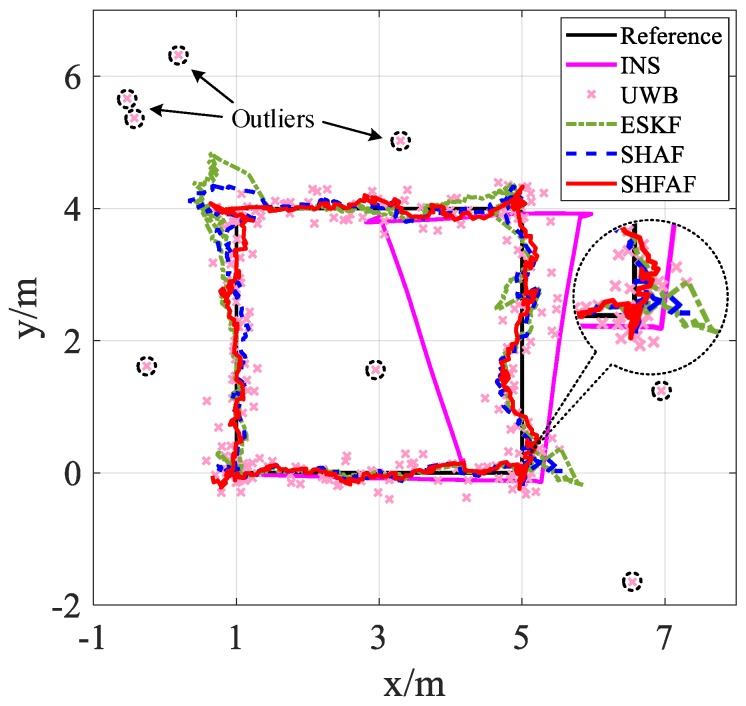
The positioning results of Trajectory B.

**Figure 14:**
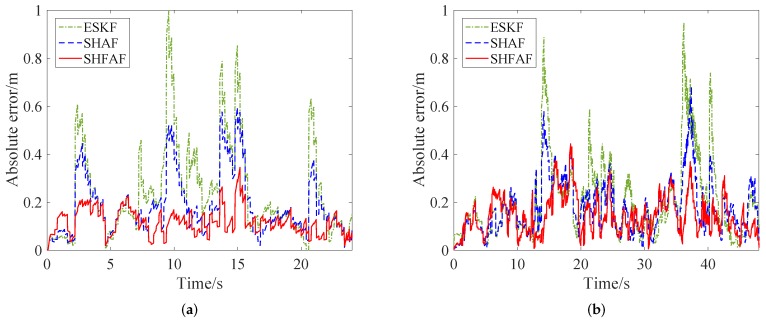
The absolute positioning errors. (**a**) Trajectory A. (**b**) Trajectory B.

As shown in Figure 12 and Figure 13, the positioning results are not consistent with the reference trajectory in the case of existing outliers. This is because UWB measurements are affected by the multipath effects and NLOS factor. However, SHFAF has the best positioning performance among several contrast approaches, which can be shown in Figure 14. Clearly, the estimate of SHFAF is closer to the truth than that of ESKF and SHAF. ESKF is greatly affected by outliers and time-varying noise, leading to a large positioning error. Although SHAF has better positioning performance than ESKF, there are still large errors of position estimation (especially in positions with abnormal measurements). Note that the experimental results of integrated positioning approaches (i.e., ESKF, SHAF and SHFAF) are better than the individual approaches including INS and UWB. In addition, the performances of these integrated positioning approaches are consistent with the simulation results in Section 4.1.

**Figure 15:**
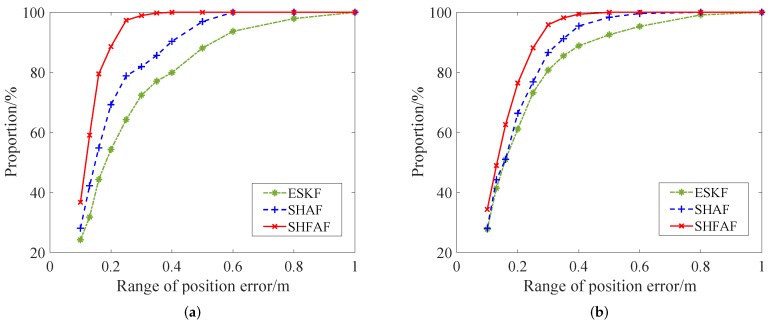
The distribution of positioning errors. (**a**) Trajectory A. (**b**) Trajectory B.

**Figure 16:**
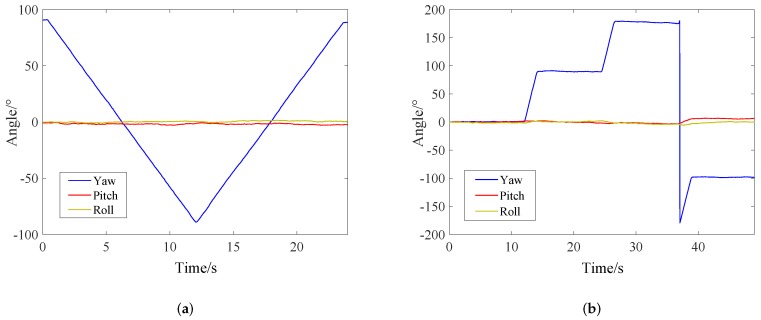
The estimated attitude angles with proposed approach. (**a**) Trajectory A. (**b**) Trajectory B.

The absolute positioning error range of trajectory A and B are shown in Figure 15. The position estimation errors less than 0.2 m of SHFAF account for 88.6% of the total sampling points in Figure 15a, while SHAF and ESKF account for 69.3% and 54.3%, respectively. From Figure 15b, we can see that the positioning results of SHFAF, SHAF and ESKF with the error less than 0.2 m stand at 88.2%, 76.8% and 61.1%, respectively. It can be concluded that the error distribution of SHFAF is more concentrated than ESKF and SHAF in a small area of less than 0.2 m. In addition, the proposed integrated positioning approach can also estimate the attitude of the robot because of using the 6-DOF kinematic model. The estimated attitude angles in this experiment are shown in Figure 16.

In summary, results and performance analyses demonstrate that SHFAF has good self-adaptive ability and robustness for applying it in the INS/UWB integrated positioning system, which satisfies the requirement on the positioning accuracy for a mobile robot in complicated indoor environments.

## 5. Conclusions

This paper focuses on the INS/UWB integrated positioning for autonomous mobile robots in complex indoor environments. To deal with the time-varying noise and outliers in UWB measurements caused by the multipath effects and NLOS factor, a robust INS/UWB integrated positioning approach is proposed based on the Sage–Husa fuzzy adaptive filter. The difficult but important problem of time-varying noise is considered explicitly in SHFAF. So in this paper, the regulatory factor is obtained by the fuzzy inference system to adjust innovation contribution weight of SHFAF adaptively, which facilitates the accurate estimation of measurement noise covariance. Specifically for outliers detection and correction, we propose an effective sliding window estimation with fading coefficients based on innovation orthogonality. As a result, the capture ability to outliers is enhanced and the accuracy of covariance estimation is guaranteed. The effectiveness of what we proposed is demonstrated through simulation and experimental results, which achieves excellent positioning performance with strong robustness for mobile robots in complicated indoor environments. Because of the limited experimental conditions, we can only evaluate the proposed INS/UWB integrated positioning approach in a regular sized laboratory. Actually, a lager experimental site should also be taken into consideration. In this context, more work remains to be done.

## Figures and Tables

**Table 1:** The root mean square error (RMSE) with different positioning approaches.

Positioning Approach	RMSE (m)		
	*East*	*North*	*Absolute*
UWB	0.5661	0.5707	0.8038
ESKF	0.2802	0.2719	0.3904
SHAF	0.2357	0.2159	0.3196
SHFAF	0.1045	0.0991	0.1440

**Table 2:** The technical specifications of sensors.

Sensors		Specification
**IMU**	**Gyroscope**	Range: ±2000${}^{\circ }$/sec Resolution: 0.07${}^{\circ }$/sec/LSB Gyro rate noise: 0.03 dps/$\sqrt{H}z$ Data bits: 16 bits
	**Accelerometer**	Range: ±16 g Resolution: 3.9 mg/LSB Impact resistance: 10,000 g Data bits: 12 bits
	**Mgnetometer**	Range: ±8 gauss Resolution: 5 milli-gauss Hysteresis: ±25 ppm Data bits: 16 bits
**UWB**		Communication rate: 110 kbit/s, 850 kbit/s, 6.8 Mbit/s Frequency: 3.5 GHz∼6.5 GHz Transmit power: −35 dbm/MHz∼62 dbm/MHz Communication distance: 30 m (Indoor), 50 m (Unobstructed) Ranging error: ±10 cm (Typical), ±30 cm (General hindered)

## References

[1] Mostafa M., Zahran S., Moussa A., El-Sheimy N., Sesay A. (2018). Radar and visual odometry integrated system aided navigation for UAVS in GNSS denied environment. Sensors.

[2] Nascimento P.P.L.L., Kimura B.Y.L., Guidoni D.L., Villas L.A. (2018). An Integrated Dead Reckoning with Cooperative Positioning Solution to Assist GPS NLOS Using Vehicular Communications. Sensors.

[3] Song Y., Nuske S., Scherer S. (2017). A multi-sensor fusion MAV state estimation from long-range stereo, IMU, GPS and barometric sensors. Sensors.

[4] Ziebold R., Medina D., Romanovas M., Lass C., Gewies S. (2018). Performance characterization of GNSS/IMU/DVL integration under real maritime jamming conditions. Sensors.

[5] Li L., Yang M., Wang C., Wang B. (2018). Hybrid Filtering Framework Based Robust Localization for Industrial Vehicles. IEEE Trans. Ind. Inform..

[6] Huang Z., Zhu J., Yang L., Xue B., Wu J., Zhao Z. (2015). Accurate 3-D Position and Orientation Method for Indoor Mobile Robot Navigation Based on Photoelectric Scanning. IEEE Trans. Instrum. Meas..

[7] Zhao S., Huang B., Liu F. (2018). Localization of Indoor Mobile Robot Using Minimum Variance Unbiased FIR Filter. IEEE Trans. Autom. Sci. Eng..

[8] Li Z., Tian Z., Zhou M., Zhang Z., Jin Y. (2018). Awareness of Line-of-Sight Propagation for Indoor Localization Using Hopkins Statistic. IEEE Sens. J..

[9] Sabet M.T., Daniali H.R., Fathi A.R., Alizadeh E. (2017). Experimental analysis of a low-cost dead reckoning navigation system for a land vehicle using a robust AHRS. Robot. Auton. Syst..

[10] Huang Y., Zhang Y., Wang X. (2017). Kalman-Filtering-Based In-Motion Coarse Alignment for Odometer-Aided SINS. IEEE Trans. Instrum. Meas..

[11] Wang C., Li K., Liang G., Chen H., Huang S., Wu X. (2017). A heterogeneous sensing system-based method for unmanned aerial vehicle indoor positioning. Sensors.

[12] Hellmers H., Kasmi Z., Norrdine A., Eichhorn A. (2018). Accurate 3D positioning for a mobile platform in non-line-of-sight scenarios based on IMU/magnetometer sensor fusion. Sensors.

[13] Xu Y., Ahn C.K., Shmaliy Y.S., Chen X., Li Y. (2018). Adaptive robust INS/UWB-integrated human tracking using UFIR filter bank. Measurement.

[14] Fan Q., Sun B., Sun Y., Zhuang X. (2017). Performance Enhancement of MEMS-Based INS/UWB Integration for Indoor Navigation Applications. IEEE Sens. J..

[15] Aditya S., Molisch A.F., Behairy H.M. (2018). A Survey on the Impact of Multipath on Wideband Time-of-Arrival Based Localization. Proc. IEEE.

[16] Davari N., Gholami A., Shabani M. (2017). Multirate Adaptive Kalman Filter for Marine Integrated Navigation System. J. Navig..

[17] Atia M.M., Liu S., Nematallah H., Karamat T.B., Noureldin A. (2015). Integrated indoor navigation system for ground vehicles with automatic 3-D alignment and position initialization. IEEE Trans. Veh. Technol..

[18] Deng Z.A., Wang G., Qin D., Na Z., Cui Y., Chen J. (2016). Continuous indoor positioning fusing WiFi, smartphone sensors and landmarks. Sensors.

[19] Jayasiri A., Nandan A., Imtiaz S., Spencer D., Islam S., Ahmed S. (2017). Dynamic Positioning of Vessels Using a UKF-Based Observer and an NMPC-Based Controller. IEEE Trans. Autom. Sci. Eng..

[20] Liu J., Cai B., Wang J. (2016). Cooperative Localization of Connected Vehicles: Integrating GNSS With DSRC Using a Robust Cubature Kalman Filter. IEEE Trans. Intell. Transp..

[21] Cui B., Chen X., Tang X. (2017). Improved Cubature Kalman Filter for GNSS/INS Based on Transformation of Posterior Sigma-Points Error. IEEE Trans. Signal Process..

[22] Kottath R., Poddar S., Das A., Kumar V. (2016). Window based Multiple Model Adaptive Estimation for Navigational Framework. Aerosp. Sci. Technol..

[23] Liu Y., Fan X., Lv C., Wu J., Li L., Ding D. (2018). An innovative information fusion method with adaptive Kalman filter for integrated INS/GPS navigation of autonomous vehicles. Mech. Syst. Signal Process..

[24] Al-Sharman M.K., Emran B.J., Jaradat M.A., Najjaran H., Al-Husari R., Zweiri Y. (2018). Precision landing using an adaptive fuzzy multi-sensor data fusion architecture. Appl. Soft Comput..

[25] Fang X., Nan L., Jiang Z., Chen L. (2017). Noise-aware fingerprint localization algorithm for wireless sensor network based on adaptive fingerprint Kalman filter. Comput. Netw..

[26] Zhong M., Guo J., Zhou D. (2018). Adaptive In-Flight Alignment of INS/GPS Systems for Aerial Mapping. IEEE Trans. Aerosp. Electron. Syst..

[27] Han H., Xu T., Wang J. (2016). Tightly coupled integration of GPS ambiguity fixed precise point positioning and MEMS-INS through a troposphere-constrained adaptive kalman filter. Sensors.

[28] Nyqvist H.E., Skoglund M.A., Hendeby G., Gustafsson F. Pose estimation using monocular vision and inertial sensors aided with ultra wide band. Proceedings of the 2015 International Conference on Indoor Positioning and Indoor Navigation (IPIN).

[29] Li Z., Chang G., Gao J., Wang J., Hernandez A. (2016). GPS/UWB/MEMS-IMU tightly coupled navigation with improved robust Kalman filter. Adv. Space Res..

[30] Wang W., Zhang B., Wang D., Jiang Y., Qin S., Xue L. (2016). Anomaly detection based on probability density function with Kullback–Leibler divergence. Signal Process..

[31] Wang L., Li S. (2018). Enhanced Multi-sensor Data Fusion Methodology based on Multiple Model Estimation for Integrated Navigation System. Int. J. Control Autom..

[32] Zhen W., Zeng S., Soberer S. Robust localization and localizability estimation with a rotating laser scanner. Proceedings of the 2017 IEEE International Conference on Robotics and Automation (ICRA).

[33] Herda L., Urtasun R., Fua P., Hanson A. (2003). Automatic Determination of Shoulder Joint Limits Using Quaternion Field Boundaries. Int. J. Robot. Res..

[34] Fang W., Zheng L., Deng H. A motion tracking method by combining the IMU and camera in mobile devices. Proceedings of the 2016 International Conference on Sensing Technology (ICST).

[35] Widy A., Woo K.T. Robust attitude estimation method for underwater vehicles with external and internal magnetic noise rejection using Adaptive Indirect Kalman Filter. Proceedings of the 2017 IEEE/RSJ International Conference on Intelligent Robots and Systems (IROS).

[36] Sun J., Xu X., Liu Y., Zhang T., Li Y. (2016). FOG random drift signal denoising based on the improved AR model and modified Sage–Husa adaptive Kalman filter. Sensors.

[37] Ke W., Wu L. (2011). Mobile location with NLOS identification and mitigation based on modified Kalman filtering. Sensors.

[38] Fan Q., Sun B., Sun Y., Wu Y., Zhuang X. (2017). Data Fusion for Indoor Mobile Robot Positioning Based on Tightly Coupled INS/UWB. J. Navig..

